# Acupuncture for nummular eczema of the lower legs in an older adult: a case report

**DOI:** 10.3389/fmed.2026.1917396

**Published:** 2026-08-14

**Authors:** Jinyuan Pan, Hongmin Yu, Wenjia Ye, Yiyun Liang

**Affiliations:** Department of Traditional Chinese Medicine, Zongrui Hospital of Beilun, Ningbo, China

**Keywords:** acupuncture, case report, nummular dermatitis, nummular eczema, older adult, pruritus

## Abstract

**Background:**

Nummular eczema, also known as nummular dermatitis or discoid eczema, is a chronic inflammatory dermatosis characterized by coin-shaped or discoid eczematous plaques with pruritus, most commonly affecting the extremities, particularly the lower legs. Persistent itch, barrier dysfunction, and repeated scratching may impair sleep and quality of life. Direct evidence for acupuncture in the treatment of nummular eczema remains limited.

**Case presentation:**

A 68-year-old woman presented with symmetrical coin-shaped erythema, scaling, and infiltrated plaques, accompanied by severe pruritus on the lateral aspects of both lower legs for more than 1 month. Previous oral doxycycline, antihistamines, and potent topical corticosteroids had provided limited benefit. At presentation, the visual analog scale (VAS) score was 8; the retrospective assessment of the baseline clinical photographs yielded an Investigator’s Global Assessment (IGA) score of 3. The clinical diagnosis was nummular eczema. She received manual acupuncture at bilateral Quchi (LI11), Xuehai (SP10), Zusanli (ST36), Yanglingquan (GB34), Zulinqi (GB41), and Xiangu (ST43), three times weekly for a total of six sessions.

**Outcomes:**

Pruritus and skin lesions improved progressively. VAS scores decreased from 8 at baseline to 3, 1, and 0 at the second, fourth, and sixth treatment visits, respectively; retrospective IGA scores based on the available clinical photographs decreased in parallel from 3 to 2, 1, and 0. By treatment completion, the bilateral lower-leg lesions had almost resolved, with only mild residual pigmentation. No clinically apparent adverse events occurred. At 4 and 12 weeks after treatment, the patient reported no obvious recurrence of pruritus or skin lesions.

**Conclusion:**

In this case, acupuncture was temporally associated with progressive relief of pruritus and near-complete resolution of nummular eczema lesions after a limited response to short-term conventional pharmacological treatment. Acupuncture may be considered as a potential adjunctive option for selected patients, but controlled studies are required to clarify its specific efficacy and safety.

## 1 Introduction

Nummular eczema, also termed nummular dermatitis or discoid eczema, is a pruritic inflammatory dermatosis characterized by round or oval, relatively well-demarcated eczematous plaques. The extremities, particularly the lower legs, are commonly affected. Although its pathogenesis remains incompletely defined, proposed contributors include xerosis, impaired epidermal barrier function, environmental or contact irritants, bacterial colonization, chronic venous stasis, medication-related factors, and immune dysregulation (1). Integrated clinical, histopathological, and transcriptomic analyses have further suggested that nummular eczema overlaps with atopic dermatitis in terms of barrier disruption and inflammatory signatures, with a codominant Th2/Th17 immune response (2).

Although nummular eczema is rarely life-threatening, persistent pruritus, recurrent lesions, and nocturnal symptoms can substantially impair sleep, mood, and quality of life. These issues are particularly relevant in older adults, who often have xerosis, reduced capacity for barrier repair, chronic comorbidities, and polypharmacy. Consequently, the prolonged use of potent topical corticosteroids or sedating systemic medications may be limited by tolerability concerns. Epidemiological data indicate that eczema is common among adults and that the prevalence varies by age and race or ethnicity (3).

The standard management of nummular eczema generally includes the identification and avoidance of aggravating factors, the regular use of emollients, and topical corticosteroids or other anti-inflammatory therapies. Antimicrobial therapy, antihistamines, phototherapy, or systemic treatment may be considered according to disease severity, infection status, and pruritus burden (1). Topical corticosteroids, calcineurin inhibitors, emollient therapy, and other barrier-restoring strategies have been systematically discussed in atopic dermatitis management guidelines (4). However, the prolonged use of potent topical corticosteroids may cause local adverse effects, including skin atrophy and telangiectasia, while some systemic medications may increase the risk of somnolence, drug interactions, and hepatic or renal burden in older adults. Safe, repeatable, and acceptable nonpharmacological interventions may therefore have clinical relevance.

Acupuncture has been used as an adjunctive intervention for several pruritic and inflammatory skin diseases. Randomized controlled trials in atopic dermatitis have suggested that acupuncture may improve symptoms in patients with mild-to-moderate disease without serious adverse events (5, 6). Systematic reviews and meta-analyses also indicate a potential benefit for atopic eczema or dermatitis, although certainty remains limited by small sample sizes, heterogeneity of interventions, and risk of bias (7, 8). Direct evidence for acupuncture in nummular eczema is scarce. We, therefore, report a case of nummular eczema in an older woman who had responded inadequately to a short course of conventional pharmacological treatment and improved during a standardized course of acupuncture (Table 1). This report was prepared with reference to the CAse REport (CARE) guidelines and the Revised STandards for Reporting Interventions in Clinical Trials of Acupuncture (STRICTA) recommendations for reporting acupuncture interventions (9, 10).

**Table 1:** Timeline of the patient’s clinical course, treatments, and outcomes.

Clinical event	Date	Treatment and duration	Clinical findings and outcomes
Symptom onset	Early March 2026	None	Symmetrical coin-shaped erythematous lesions with pruritus appeared on the lateral aspects of both lower legs. The lesions gradually increased in number and size, and nocturnal pruritus worsened and interfered with sleep.
Pharmacological treatment at an outside hospital	10 March 2026	Oral doxycycline hydrochloride 100 mg once daily; oral desloratadine citrate disodium 8.8 mg once daily; topical halometasone cream.	No substantial improvement in pruritus or lesion extent.
Pharmacological treatment at an outside hospital	20 March 2026	Oral ebastine 10 mg once daily; oral cetirizine hydrochloride 10 mg once daily; topical compound clobetasol propionate ointment (0.05% clobetasol propionate and 0.025% tretinoin).	Pruritus improved slightly, but nocturnal pruritus remained poorly controlled; neither lesion extent nor infiltration improved meaningfully.
Pharmacological treatment at an outside hospital	3 April 2026	Oral ebastine 10 mg once daily; oral cetirizine hydrochloride 10 mg once daily; topical halometasone cream.	Pruritus improved slightly, but nocturnal pruritus remained poorly controlled; neither lesion extent nor infiltration improved meaningfully.
Acupuncture treatment at our department	14–28 April 2026	Manual acupuncture three times weekly for six sessions.	Pruritus progressively resolved, and the lesions gradually subsided, leaving only mild residual pigmentation and a smoother skin surface.
First follow-up	26 May 2026	No additional treatment.	No obvious recurrence of pruritus or skin lesions was reported.
Second follow-up	21 July 2026	No additional treatment.	No obvious recurrence of pruritus or skin lesions was reported.

## 2 Case presentation

A 68-year-old woman presented to the Department of Traditional Chinese Medicine on 14 April 2026, with coin-shaped skin lesions on the lateral aspects of both lower legs accompanied by severe pruritus that had persisted for more than 1 month. She denied a history of hypertension, diabetes mellitus, allergic disease, or other chronic conditions and reported no long-term medication use other than treatment for the current skin condition. She did not smoke or consume alcohol. There was no family history of eczema, atopic dermatitis, psoriasis, or other skin disease, and she reported no specific occupational exposure or psychosocial stressors. More than 1 month before presentation, symmetrical round erythematous lesions had appeared on the lateral lower legs without an obvious trigger. The lesions were initially limited but gradually increased in number and size. Pruritus was persistent, worsened at night, and interfered with both sleep onset and sleep maintenance. Over the course of the disease, the lesions became dry, scaly, and locally infiltrated. Scratching provided only transient relief, followed by recurrent itching. The patient denied obvious erosion, exudation, bleeding, fever, or other systemic symptoms.

Before presentation to our department, the patient had been treated at another dermatology department. She initially received oral doxycycline hydrochloride, desloratadine citrate disodium, and topical halometasone cream for 10 days, but neither pruritus nor the skin lesions improved substantially. Treatment was then changed to oral ebastine and cetirizine hydrochloride, with compound clobetasol propionate ointment followed by halometasone cream, for more than 20 days. Daytime pruritus improved only slightly, whereas nocturnal pruritus remained poorly controlled; neither lesion extent nor infiltration showed clinically meaningful improvement. Because of the limited response to short-term conventional treatment and the persistent impact of pruritus on sleep and daily life, the patient requested acupuncture treatment.

Dermatological examination revealed multiple round-to-oval, coin-shaped erythematous and infiltrated plaques on the lateral aspects of both lower legs. The lesions were relatively well demarcated, varied in size, and measured approximately 1.5–3.0 cm in diameter, with symmetrical bilateral distribution. The lesion surfaces were dry and finely scaly; some areas were mildly thickened and lichenified. There was no obvious exudation, erosion, crusting, pustulation, ulceration, secondary infection, or lower-limb edema. Sensory and motor function in both lower limbs was normal, and no obvious neurological abnormality was observed. Baseline pruritus intensity, assessed using a visual analog scale (VAS), was 8, indicating severe itching. The retrospective assessment of the baseline clinical photographs using the Investigator’s Global Assessment (IGA) yielded a score of 3, indicating moderate overall lesion severity with definite erythema, scaling, and infiltration. The VAS is a widely used patient-reported outcome measure for assessing pruritus intensity and treatment-related symptom change (11). The IGA is a widely used investigator-reported outcome in dermatology clinical trials for grading overall lesion severity (12).

A clinical diagnosis of nummular eczema was made on the basis of the symmetrical coin-shaped erythematous and infiltrated plaques on both lower legs, severe pruritus, xerosis and scaling, and the absence of obvious exudation, erosion, crusting, pustulation, fever, or other signs of acute infection. Differential diagnoses included tinea corporis, allergic contact dermatitis, stasis dermatitis, psoriasis, and cutaneous T-cell lymphoma (1). At the initial assessment, the lesion morphology and distribution were considered consistent with nummular eczema, and there were no features strongly suggestive of unilateral progressive disease, marked exudation or ulceration, lower-limb edema, thick psoriasiform plaques, or systemic illness. Fungal microscopy or culture, patch testing, skin biopsy, and laboratory tests were therefore not performed. This lack of ancillary testing limits diagnostic certainty, and the exclusion of mimicking conditions was based primarily on clinical judgment. In patients with atypical morphology, unilateral distribution, disease recurrence, marked exudation or ulceration, lower-limb edema, or persistent non-response to conventional therapy, ancillary investigations should be considered.

In traditional Chinese medicine terms, the patient had red eruptions, severe pruritus, a red tongue with a thin yellow coating, and a thin, wiry, forceful pulse. Considering the lower-limb distribution and the disease course, the pattern was interpreted as heat in the blood aspect combined with downward diffusion of damp-heat, resulting in pruritus.

## 3 Therapeutic intervention

Before treatment, the physician explained the treatment plan, expected benefits, potential risks, and skin self-care measures, and the patient provided written informed consent. All acupuncture procedures were performed by a chief physician with more than 40 years of clinical acupuncture experience. The patient was placed in the supine position. Bilateral Quchi (LI11), Xuehai (SP10), Zusanli (ST36), Yanglingquan (GB34), Zulinqi (GB41), and Xiangu (ST43) acupoints were selected (Table 2).

**Table 2:** Acupoints, anatomical locations, and needle insertion depths.

Acupoint	Anatomical location	Insertion depth (mm)
Quchi (LI11)	At the elbow region, in the depression at the midpoint of the line connecting Chize (LU5) and the lateral epicondyle of the humerus.	30–40
Xuehai (SP10)	On the anterior aspect of the thigh, 2 cun proximal to the medial end of the base of the patella, on the bulge of the vastus medialis muscle.	30–40
Zusanli (ST36)	On the lateral aspect of the lower leg, 3 cun inferior to Dubi and one finger-breadth lateral to the anterior crest of the tibia.	30–40
Yanglingquan (GB34)	On the lateral aspect of the lower leg, in the depression anterior and inferior to the head of the fibula.	30–40
Zulinqi (GB41)	On the dorsum of the foot, between the fourth and fifth metatarsal bones, in the depression proximal to the fourth metatarsophalangeal joint.	20–25
Xiangu (ST43)	On the dorsum of the foot, between the second and third metatarsal bones, in the depression proximal to the second metatarsophalangeal joint.	20–25

The acupoint prescription was based on both traditional syndrome differentiation and lesion distribution. Quchi and Xuehai were selected as principal acupoints to clear heat, regulate blood, dispel wind, and relieve itching. Because the lesions were located primarily on the lateral aspects of both lower legs, corresponding broadly to the courses of the Foot Yangming Stomach and Foot Shaoyang Gallbladder meridians, Zusanli, Xiangu, Yanglingquan, and Zulinqi were added to regulate the local circulation of qi and blood and to clear downward damp-heat. This rationale was derived from traditional acupuncture theory and was used to guide the acupuncture regimen.

The skin over each acupoint was disinfected with alcohol before needling. Disposable sterile stainless-steel Huatuo-brand acupuncture needles were used, including 0.30 mm × 40 mm and 0.30 mm × 25 mm needles. For Quchi, Xuehai, Zusanli, and Yanglingquan, 0.30 mm × 40 mm needles were inserted perpendicularly to a depth of approximately 30–40 mm. For Zulinqi and Xiangu, 0.30 mm × 25 mm needles were inserted perpendicularly to a depth of approximately 20–25 mm. After insertion, manual stimulation was applied until de qi was achieved, mainly perceived as local soreness, distension, or heaviness. Needles were retained for 30 min. During retention, manual stimulation was performed once every 10 min with bilateral alternating rotation, an amplitude of approximately 180°, and a frequency of approximately 60 rotations per minute. After needle removal, local pressure was applied to prevent bleeding. Treatment was administered three times per week for a total of six sessions.

During the acupuncture treatment period, the patient discontinued her previous oral and topical medications. The physician also provided basic skin-care advice, including avoidance of scratching, hot-water washing, alkaline cleansers, wool or other irritating fabrics, and suspected contact irritants. The patient was advised to cleanse the skin gently with warm water and to use emollients regularly.

## 4 Follow-up and outcomes

After the first acupuncture session, the patient reported noticeable relief of pruritus. Over the treatment course, both pruritus severity and skin lesions progressively improved. At the second treatment visit, erythema and scaling on the lateral aspects of both lower legs were slightly reduced, nocturnal pruritus was markedly relieved, and the VAS score decreased from 8 at baseline to 3. At the fourth treatment visit, pruritus improved further and the VAS score decreased to 1; the bilateral coin-shaped erythematous lesions had decreased in extent and intensity, with reduced plaque infiltration and scaling. At the sixth treatment visit, the patient reported complete resolution of pruritus and a VAS score of 0; the bilateral lower-leg lesions had almost completely resolved, leaving only mild residual pigmentation and a smoother skin surface. Retrospective IGA scores based on the available clinical photographs decreased from 3 at baseline to 2, 1, and 0 at the second, fourth, and sixth treatment visits, respectively.

**Figure 1:**
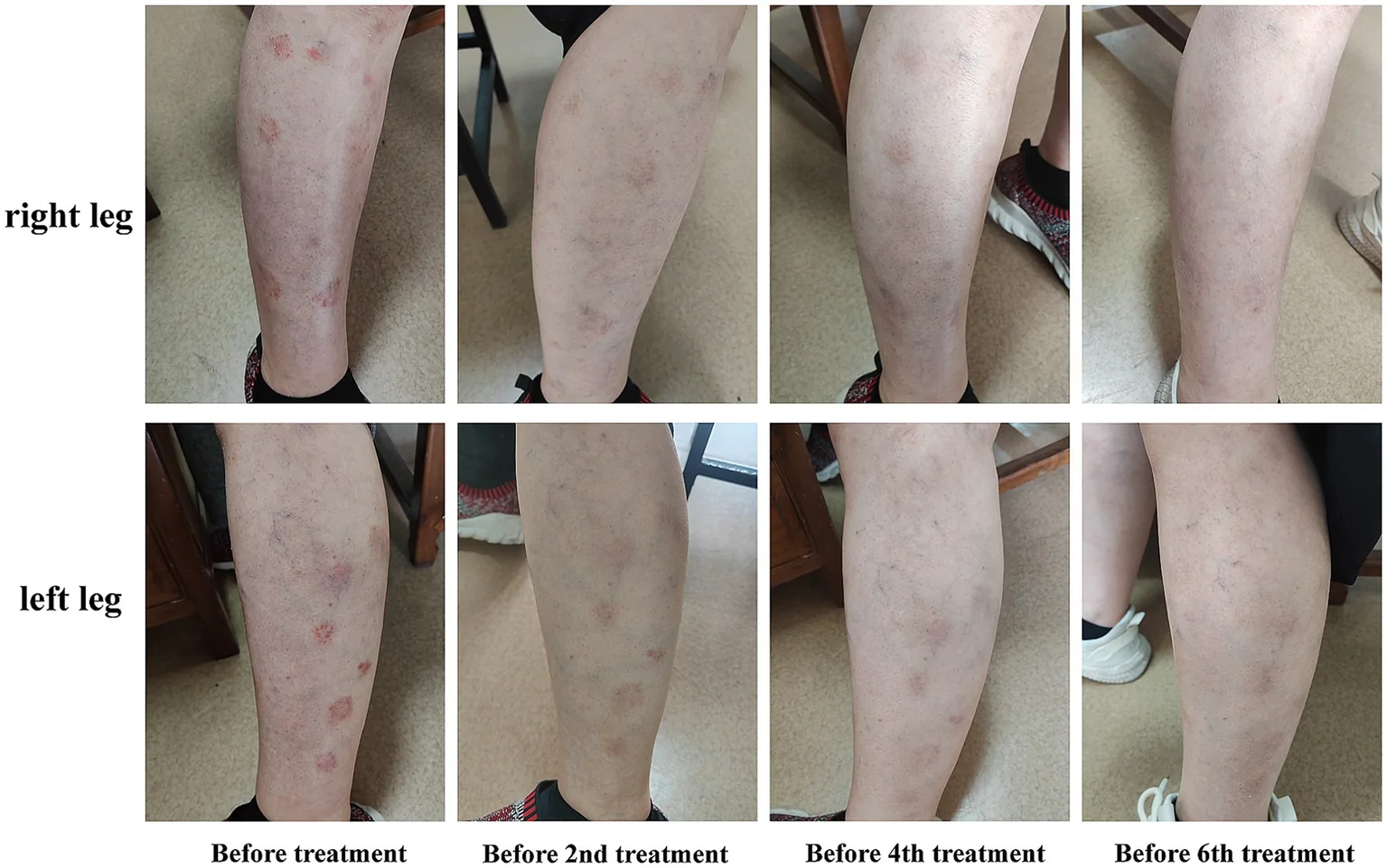
Progressive improvement in bilateral lower-leg lesions during six sessions of acupuncture.

**Figure 2:**
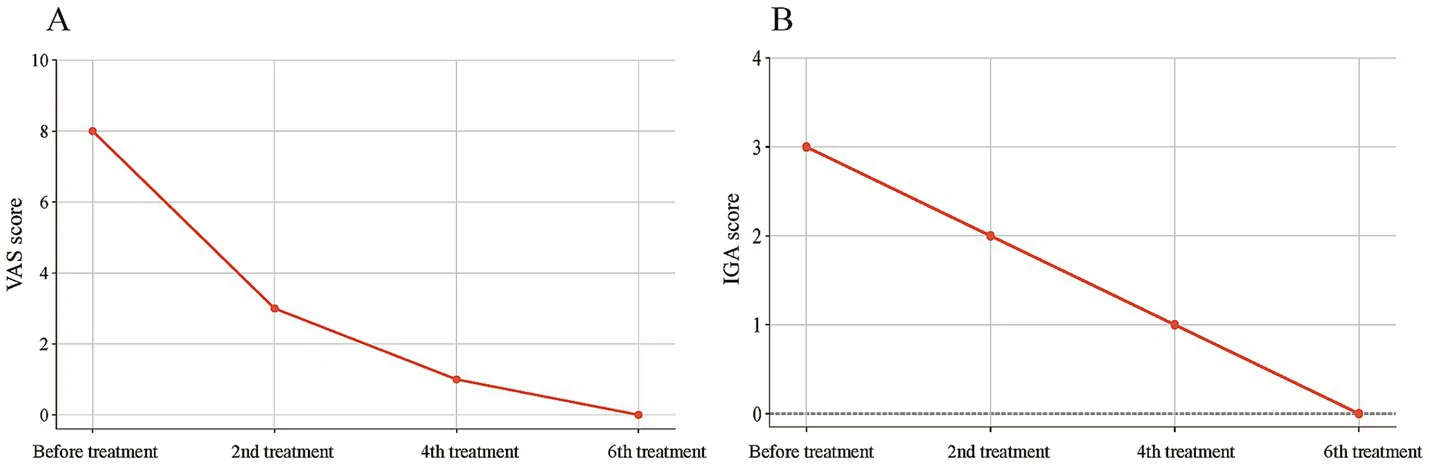
Changes in pruritus severity and overall lesion severity during six acupuncture sessions **(A)** VAS; 0–10 scores decreased from 8 at baseline to 3, 1, and 0 at the second, fourth, and sixth treatment visits, respectively. **(B)** IGA scores decreased in parallel from 3 to 2, 1, and 0.

No fainting, local infection, persistent bleeding, marked pain, hematoma, worsening of skin lesions, or other adverse events occurred during treatment. At the 4- and 12-week telephone follow-ups, the patient reported no obvious recurrence of pruritus or skin lesions, and sleep quality had improved compared with baseline. Changes in the skin lesions and in the VAS and IGA scores during treatment are shown in Figures 1, 2, respectively.

## 5 Discussion

The main therapeutic challenge in nummular eczema is the interaction among cutaneous inflammation, barrier dysfunction, and the itch-scratch cycle. The condition may be associated with xerosis, contact or environmental irritation, microbial colonization, venous stasis, and immune abnormalities (1). Recent molecular evidence further suggests that nummular eczema and atopic dermatitis are not entirely independent disease entities; instead, they share features of barrier impairment and immune inflammation, and nummular eczema may show a combined Th2/Th17 inflammatory profile (2). In the present case, the patient was an older woman with symmetrical coin-shaped erythematous, scaly, infiltrated plaques on the lateral aspects of both lower legs, accompanied by persistent nocturnal pruritus. In older adults, xerosis and reduced barrier-repair capacity are common. Repeated scratching may further disrupt the stratum corneum and amplify local inflammation, thereby sustaining an itch-scratch-inflammation cycle. Studies of itch mechanisms indicate close interactions among keratinocytes, immune cells, mast cells, and peripheral sensory nerves; chronic itch may not only be a symptom of cutaneous inflammation but also contribute to its persistence (13, 14). The early relief of pruritus before the complete resolution of skin lesions in this patient is therefore clinically relevant: Reduced itching may have decreased scratching and created more favorable conditions for barrier recovery and resolution of inflammation.

The current management of nummular eczema usually involves avoidance of aggravating factors, regular emollient use, topical corticosteroids, and, when necessary, antimicrobial therapy, antihistamines, phototherapy, or systemic treatment (1, 4). Before presentation to our department, this patient had received oral antihistamines, oral doxycycline, and potent topical corticosteroids, but pruritus and skin lesions showed limited improvement. After initiation of acupuncture, the pruritus VAS score decreased from 8 at baseline to 3, 1, and 0 at the second, fourth, and sixth treatment visits, respectively; IGA scores similarly decreased from 3 to 2, 1, and 0. In parallel, the coin-shaped erythema, scaling, and plaque infiltration on both lower legs gradually improved, and the lesions had almost resolved by the sixth session. These changes indicate a temporal association between acupuncture treatment and symptom improvement in this case. For selected patients with an inadequate response to conventional treatment, sleep disturbance due to pruritus, or a desire to reduce medication burden, acupuncture may have value as an adjunctive therapeutic option.

The mechanisms by which acupuncture may influence eczema-associated pruritus remain incompletely understood. Basic and review studies suggest that acupuncture may affect inflammation and itch perception through neuroimmune regulation, modulation of peripheral inflammatory mediator release, autonomic reflexes, the hypothalamic–pituitary–adrenal axis, and central itch-processing networks (15–17). These mechanisms are relevant to eczema because inflammatory mediators, neural sensitization, and scratching behavior may reinforce one another. Chronic pruritus often involves non-histaminergic neural pathways and immune–neural interactions, which may help explain why some patients respond insufficiently to antihistamines yet improve after interventions that modulate neural function (18). These neuroimmune mechanisms remain hypothetical in the context of this case report and require confirmation in future mechanistic and controlled clinical studies. Moreover, skin barrier function, cytokines, microbial colonization, and neural biomarkers were not assessed in this patient. Therefore, no conclusion can be drawn regarding a specific biological mechanism of acupuncture in this case.

Clinical evidence for acupuncture in eczema is currently concentrated mainly in atopic dermatitis. Previous randomized or sham-controlled trials have reported improvements in pruritus, skin lesions, or quality-of-life measures among patients with mild-to-moderate atopic dermatitis (5, 6). Systematic reviews and meta-analyses also suggest a potential benefit of acupuncture for atopic eczema or dermatitis; however, the available studies remain limited by small sample sizes, heterogeneous interventions, difficulty with blinding, and risk of bias (7, 8). Direct evidence for acupuncture in nummular eczema is much more limited. A previous case report of refractory nummular eczema described improvement in pruritus, anxiety, and dermatology-related quality of life after local shallow needling, surrounding needling, and meridian acupuncture (19). The present case differs from that report in several respects: the patient was older, the lesions were bilaterally distributed on the lower limbs, and the regimen primarily used distal or non-lesional acupoints, including Quchi, Xuehai, Zusanli, Yanglingquan, Zulinqi, and Xiangu, rather than dense needling around active eczematous plaques. This approach may be more suitable for patients in whom local stimulation of active lesions raises concerns about irritation, bleeding, or infection, and it provides another case-level observation of acupuncture treatment for nummular eczema.

From the perspective of intervention design, the acupoint prescription combined traditional syndrome differentiation with lesion distribution. Quchi (LI11) and Xuehai (SP10) were selected as principal points to clear heat, cool and regulate blood, and relieve itching. Zusanli (ST36), Yanglingquan (GB34), Zulinqi (GB41), and Xiangu (ST43) were selected in relation to lower-limb lesion distribution, the courses of the Foot Yangming Stomach and Foot Shaoyang Gallbladder meridians, and the traditional pattern interpretation of heat in the blood aspect with downward damp-heat. Compared with case reports that describe acupuncture only in general terms, this report provides details on needle specifications, insertion depth, de qi, retention time, manipulation frequency, treatment frequency, and concomitant skin-care measures, consistent with the STRICTA recommendations for reproducible reporting of acupuncture interventions (10). No fainting, infection, persistent bleeding, marked pain, hematoma, or worsening of skin lesions occurred during treatment, suggesting good tolerability in this patient. Nevertheless, this was a single case report, and the 12-week follow-up was insufficient to assess long-term recurrence. Natural remission, delayed effects of previous medications, and the effects of basic skin care cannot be excluded. The IGA was assessed retrospectively from clinical photographs and may therefore be subject to observer bias; moreover, the IGA framework has not been validated specifically for nummular eczema. Accordingly, more rigorously designed clinical studies are needed to evaluate the efficacy of acupuncture for nummular eczema.

## 6 Patient perspective

“My lower legs had been itching for more than a month, and I could not sleep well at night. The ointments and tablets did not work very well. After several acupuncture sessions, the itching was clearly relieved and the redness gradually faded. After six sessions, the skin had basically recovered, with only slight color changes, and there was no recurrence through the 12-week follow-up. My sleep and quality of life improved.”

## 7 Conclusion

In this case, acupuncture was temporally associated with progressive relief of pruritus and near-complete resolution of bilateral lower-leg lesions in an older woman with nummular eczema that had shown limited improvement after a short course of conventional pharmacological treatment. Acupuncture may be considered a potential adjunctive option for selected patients who prefer nonpharmacological approaches or have limitations related to medication use. Because this report describes a single patient, the efficacy and safety of acupuncture for nummular eczema require confirmation in larger, rigorously designed clinical studies.

## Data Availability

The original contributions presented in the study are included in the article/supplementary material; further inquiries can be directed to the corresponding author.
